# Forensic Value of Genetic Variants Associated with Anti-Social Behavior

**DOI:** 10.3390/diagnostics11122386

**Published:** 2021-12-17

**Authors:** Antonio Oliva, Simone Grassi, Massimo Zedda, Marco Molinari, Stefano Ferracuti

**Affiliations:** 1 Department of Health Surveillance and Bioethics, Section of Legal Medicine, Fondazione Policlinico A. Gemelli IRCCS, Università Cattolica del Sacro Cuore, Largo Francesco Vito 1, 00168 Rome, Italy; antonio.oliva@unicatt.it (A.O.); massimo_zedda@icloud.com (M.Z.); 2 Neuro-Robot Rehabilitation Lab, IRCCS Fondazione Santa Lucia, Via Ardeatina 306, 00179 Rome, Italy; m.molinari@hsantalucia.it; 3 Department of Human Neuroscience, Sapienza University, Piazzale Aldo Moro 5, 00165 Rome, Italy; stefano.ferracuti@uniroma1.it

**Keywords:** anti-social behavior, genetic predisposition, behavioral genetics, crime, legal capacity

## Abstract

Insanity defense is sometimes invoked in criminal cases, and its demonstration is usually based on a multifactorial contribution of behavioural, clinical, and neurological elements. Neuroradiological evidence of structural alterations in cerebral areas that involve decision-making and moral reasoning is often accepted as a useful tool in these evaluations. On the other hand, the genetic predisposition to anti-social behavior is still controversial. In this paper, we describe two cases of violent crimes committed by young carriers of genetic variants associated with personality disorder; both the defendants claimed to be insane at the time of the crime. We discuss these cases and review the scientific literature regarding the relationship between legal incapacity/predisposition to criminal behavior and genetic mutations. In conclusion, despite some genetic variants being able to influence several cognitive processes (like moral judgement and impulse control), there is currently no evidence that carriers of these mutations are, per se, incapable of intentionally committing crimes.

## 1. Introduction

In criminal cases, a defense based on proving the insanity of the defendant at the time of the offence and thus the lack of his intent to commit the crime (also called in the United States “mens rea”) can be invoked. The defendant can be found not guilty for reasons of insanity only if the mental incompetence caused by severely impairing neurobiological anomalies or disorders (e.g., schizophrenia) is proved, and there is a causal link between the criminal behavior and the mental illness. In the United States, neurobiological evidence is presented with this purpose in approximately 5–6% of murder trials and 25% of death penalty trials [1]. In these cases, a multifactorial contribution of genetic, neurological and environmental factors is often invoked [1]. In the United States, in about 15% of the judicial opinions in which neurobiological evidence is discussed, the defendant undergoes neuroradiological examination (about a fourth of these tests are performed through MRI or CT) [1]. Structural and/or functional anomalies can cause severe behavioral impairment that, in some cases, may can be considered to be a legal excuse. For example, structural alterations and functional impairments of prefrontal cortex (PFC) have been associated with antisocial and criminal behavior [2]. In particular, Choy et al. studied the influence of PFC activity on criminal behavior, finding that an increase of the perceptions of moral wrongfulness in aggressive acts could be achieved through transcranial electric stimulation [3]. Moreover, psychopathic offenders tend to have a reduced grey matter volume in the prefrontal and temporal cortex [4]. In adulthood, reduced amygdala volume has also been associated with increased risk of antisocial behavior [5]. Hence, proving congenital or acquired (e.g., substances-related) structural alterations of the brain that can severely impair the intent or knowledge of wrongdoing has a strong legal impact and scientific basis. On the other hand, the genetic predisposition to anti-social behavior is controversial [6,7,8,9,10,11].

In this paper, we describe two cases of violent crimes committed by young carriers of genetic variants associated with antisocial behavior. Both the defendants were affected by personality disorders and invoked an insanity defense. Our aim is to discuss these cases and review the scientific literature regarding the relationship between legal incapacity/predisposition to criminal behavior and genetic mutations.

## 2. Cases

### 2.1. Case 1

A young man was killed by a 30-year-old man after they had consumed alcohol and cocaine. The murderer claimed he was not capable when he committed the crime because he suffered from an alcohol-/drug-caused behavioral impairment producing neurological damage, having regularly consumed alcohol and drugs since the beginning of adolescence. In detail, he reported to have started habitually consuming alcohol, cannabis, cocaine and amphetamine when he was a teenager. The defendant also claimed to be predisposed to anti-social behavior because of genetic factors. Indeed, his forensic consultant performed a genetic testing on him focused on three genes (*MAOA*, *COMT*, *SLC6A4*), finding that he was a carrier of the polymorphisms of *5-HTTLPR* (fragment 44 bp–SS genotype) and *COMT* (Leu136Leu) in homozygosity.

Hence, the court requested a team of forensic experts to assess the capacity of the defendant, performing toxicology testing and a complete neuropsychiatric evaluation.

#### 2.1.1. Toxicology Testing

Toxicology testing was performed on urine (four days after the murder), saliva (two days after the murder), blood and pubic hair (10 days after the murder). In blood and saliva, it failed to find significant levels of drugs or alcohol, while in urine it detected benzoylecgoine (322 ng/mL). In the pubic hair, significant levels of cocaine (141 ng/mg), benzoylecgonine (21 ng/mg), and ethylglucuronide in concentration >30 pg/mg were found.

#### 2.1.2. Clinical and Neuroradiological Evaluation

A full clinical/neuropsychological examination was performed. No clinical signs of neurological impairment and no signs of alcohol-dependence were observed. A personality disorder not otherwise specified was diagnosed. 3-Tesla brain MRI and brain CT-PET were also performed. In MRI imaging, a decrease in cortical thickness with larger lateral ventricles, a statistically significant volumetric asymmetry of the amygdalae (the right amygdala was smaller than the left one) and a decreased volume of the right orbito-frontal cortex (OFC) (in comparison with the left one) were observed. No ischemic lesion or anomalies in the corona radiata and in the subtentorial/cerebellar area were found. PET-CT did not find any alteration of brain perfusion or metabolism.

### 2.2. Case 2

A 25-year-old man abducted, raped and robbed two women under the influence of alcohol in six months. He reported that his father often physically and psychologically abused him and his mother during his childhood and that a teenager raped him when he was a child. He was unschooled and few years before the rapes he was convicted for having stabbed a man who had insulted him. After having been released, he committed several burglaries. Moreover, he reported to have frequently beaten his wife and to have often fantasized about raping women since he was very young, even if he knew rape was illegal. Finally, he reported to have begun to consume alcohol during his childhood, albeit he never became an alcoholic.

Hence, the court requested a forensic psychiatrist to assess the capacity of the defendant.

#### Clinical Evaluation and Genetic Testing

A full clinical/neuropsychological examination was performed. No clinical/electroencephalographical signs of neurological impairment and no signs of alcohol-dependence were observed. An intelligence quotient (IQ) of 59 was found and an antisocial personality disorder was diagnosed. A genetic test focused on five genes (*MAOA*, *COMT*, *SLC6A4*, *HTR1B*, and *DRD4*) found a 3-repeat variable number of tandem repeats (VNTR) variant of *MAOA* and a TT genotype for the rs13212041 polymorphism of the *HTR1B* gene.

## 3. Literature Review Methodology

The aim of the review was to describe an overview of the available research evidence regarding the interpretation of the significance of genetic variants in the criminal justice system, focusing on the behavioral aspects. Two investigators searched published studies through the electronic database MEDLINE via PubMed. Two search strings were used, combining couples of keywords through the Boolean operator AND: (1) “(gene) AND (criminal justice)” and (2) “(behavioral genetics) AND (criminal justice)”. The eligibility criteria were: publication date between 1 January 1995 and 12 December 2021; English language. The first search string produced 231 results, while the second search string produced 128 results. The results were compared, finding 50 overlapping papers. The investigators examined the titles and abstracts of the 309 papers, excluding 177 results because they did not fall within the review objective. Therefore, the full texts of the remaining papers were carefully read. Nine papers were excluded because they were not written in English. The final output of the review process consisted of 82 papers (cited in the bibliography).

## 4. Discussion

Case 1 committed a murder under the influence of cocaine and alcohol and defended himself claiming to be incapable of his actions because of a long history of alcohol and drug abuse which started at a very young age. No elements of toxicological, clinical or radiological signs supported the thesis of the defendant, concluding that he was affected by a personality disorder not otherwise specified.

Case 2 abducted, raped and robbed two women under the influence of alcohol and claimed to be incapable of committing the crimes. He neither reported nor showed signs of alcohol dependence and was found to be affected by an antisocial personality disorder.

Under Italian law, having committed a crime under the influence of drugs and/or alcohol does not affect legal capacity unless it is proven that the chronic consumption of illicit substances completely impaired his intent or knowledge of wrongdoing because of a severe organic dysfunction (art. 95 of the Italian criminal code). More specifically, the defendant can be found not responsible only if he proves that his mental state and/or his congenital/acquired behavioral anomalies annihilated the capacity of intending his actions. Furthermore, if the mental impairment was due to the intake of alcohol or illicit drugs, it must also be proved that the substances caused severe chronic structural damage. Hence, being the defendants theses of the described cases based only on their genetic predisposition to anti-social behavior, they were judged legally competent. Both the cases presented no signs of chronic brain damage caused by alcohol or drugs, thus their capacity to control their impulses and understand the significance and the consequences of their actions were considered substantially intact. Moreover, Case 2 had a personal history of physical, psychological and sexual abuse during childhood and, when he committed crimes, showed patterns of conscious criminal intent (e.g., he abducted the victims to rape them without being interrupted and then he took their valuables). Therefore, in Case 2 the role of genetic factors was considered marginal at most, even if the subject had a combination of polymorphisms that is described as a predisposing factor for violent and impulsive behavior.

Farahani et al. observed that in these cases the main limitation of neurobiological evidence, and in particular of genetic evidence, is represented by the fact that, while these investigations are relatively expensive, their influence over the judge’s decision is unpredictable [1]. In particular, a line of defense only based on genetic evidence is rarely used because it is almost always unsuccessful [12,13]. On the contrary, the combination of genetic evidence and environmental factors predisposing to anti-social behavior (like maltreatment, maternal disengagement and poor school performance during childhood and drug abuse) can lead to a recognition of mitigating circumstances [14,15,16,17,18]. The supporters of behavioral genetics often observe that there is also a sort a familiarity for anti-social behavior and that there are congenital anomalies that seem to predispose to criminal activities. Regarding the role of family environment, many authors reported that the criminal behavior of the parents is associated with a higher risk of their children committing crimes, even if they are adopted by parents without a criminal record [19,20]. For example, Beaver reported that the adoptees with at least a biological parent who had been arrested were at higher risk of committing crimes (OR if only a parent had been arrested: 3.25–6.58; OR if both parents had been arrested 4.73–12.87) [19]. Moreover, Wertz et al. found that monozygotic twins were more similar in their criminal offending than dizygotic twins [21]. Regarding congenital structural alterations, anomalies of prefrontal cortex, amygdala and striatum are considered to be potentially involved in criminal behavior, and some genetic variants have been associated with these alterations (for example, low *MAOA* expression has been associated with a reduction in the size of limbic system) [2,22]. However, this kind of genetic polymorphism does not always correspond to structural anomalies.

### 4.1. MAOA

The *MAOA* gene (also called the “warrior gene”) encodes the mono-amine oxidase A, that deactivates monoamine neurotransmitters like dopamine, norepinephrine and serotonin [8,23]. *MAOA* is highly expressed in PFC and under expressed in the hypothalamus [22,24]. The low-activity 2- or 3-repeat variants of *MAOA* are known as *MAOA-L* and have been associated with impulsivity and antisocial behavior [8]. In particular, carriers of *MAOA-L* often fail to manage strong emotional impulses [21]. Stetler et al. found a statistically significant association between *MAOA-L* VNTR alleles and violent crime in Caucasians (*p* < 0.01) but not in African Americans (*p* = 0.08) [25]. Since the *MAOA* gene is in chromosome X, supposedly women are less inclined to antisocial behavior [22]. According to Caspi et al., children with a history of abuse and *MAOA-H* (high activity) genotype had a lower risk of developing antisocial behavior [7]. Despite this, the long four repeat allele of *MAOA* gene (*MAOA-H*) is associated with criminal behavior in female adolescents with psychosocial risk [26]. This is a controversial topic because in women one of the two X chromosomes (and then one of the MAOA allele) is inactivated and therefore in heterozygotes can be difficult to associate the phenotype with the exact genotype [27].

Armstrong et al. found an association between *MAOA-L* genotype and criminal behavior (*p* = 0.038) and between *MAOAL* genotype and property crime arrest (*p* = 0.039) [28]. Beaver et al. reported that *MAOA-L* can increase the risk of becoming a gang member and weapon use by, respectively, 1.94 times and 1.82 times [29]. The interaction between *MAOA-L* and family environment has been studied by many authors. For example, Gonzalez-Tapia et al. found that carriers of *MAOA-L* who were victims of childhood maltreatment tended to show a reduction of PFC volume [8]. Fergusson et al. reported that in *MAOA-L* carriers there can be a relationship between criminal behavior, low IQ and family-related factors like maternal smocking during the pregnancy and childhood maltreatment [30]. On the basis of the different number of repeats, *MAOA-L* may have different effects. The 2-repeat allele of *MAOA-L* was associated with shooting and stabling (*p* = 0.05) and with the total number of shooting and stabling accidents (*p* = 0.05) [31]. In heroin addicts, the frequency of low activity 3-repeats *MAOA-L* allele was significantly higher in violent offenders (*p* = 0.03) [32]. Violent antisocial offenders had more frequently the 3-repeat allele of *MAOA-L* (*p* = 0.05) [32]. While most of the evidence is focused on *MAOA-L*, even the high-activity variants of *MAOA* have been associated with anti-social behavior. For example, the high-activity 4-repeat allele is reportedly more frequent in heroin addicts without a history of violence or crime (*p* = 0.02) [32]. Moreover, high-activity variants can increase the risk of fraudulent behavior in young males with several delinquent peers [33]. Finally, it should be noted that some authors confuted the hypothesis of the criminogenic role of *MAOA*. For example, Lu-Menard et al. did not find a clear and statistically significant relationship between *MAOA-L* and delinquent peer affiliation or criminal conduct [6].

### 4.2. 5-HTT

The serotoninergic system plays a major role in the regulation of emotions such as fear, anger and anxiety [34]. Toshchakova et al. found a statistically significant association between criminal behavior and polymorphisms of *5HTTLPR* (a region inside the gene–SLC6A4–that codes for serotonin transporter) (*p* = 0.004) and *5HTR2C* (that codes for a subtype of serotonin receptor) (*p* = 0.0026) [35]. Liao et al. have found a statistically significant association between violent behavior and the presence of a low-activity short allele of *5HTTLPR* (*p* = 0.006) [36]. In neglected females, the homozygosity for the short allele of *5-HTTLPR* has also been associated with an increased risk of cannabis abuse [37]. The SS (short-short) allele of *5HTTLPR* has been associated with aggressive behavior and mood disorders [38]. Gerra et al. found that, in heroin-addicted males, the SS allele is overexpressed and associated with violent behavior (*p* = 0.02) [39]. Even the LL (long-long) variant of *5HTTLPR* has been reported to increase the risk of criminal behavior (in males with low socioeconomic status) [40]. Berggard et al. studied the *5-HT2A-1438 GA* polymorphism in a sample of 97 Swedish imprisoned criminal men and 202 non-criminal controls. The authors found a lower rate of the *5-HT2A -1438 GG* genotype in the criminal group (*p* = 0.034) [41]. The *HTR1B* gene codes for the serotonin receptor 1B, that inhibits the release of 5-HTT in the synapsis [42]. Pauwels et al. found that *HTR1B* gene can interact with dopaminergic, glutamatergic, gamma-aminobutyric and cholinergic receptors [43]. The interaction between microRNA and specific DNA sequences can modulate the gene expression. Some studies found a sequence in the 3′UTR of *HTR1B* gene (rs13212041) that promotes the link with a specific microRNA (miR-96), and this interaction seems to be associated with violent behavior [44,45]. Furthermore, the same authors found that the TT genotype of rs13212041 polymorphism was related to anger and hostility [44,45].

### 4.3. COMT

The dopamine system is regulated by different catabolic enzymes including the catechol-O methyltransferase (encoded by the *COMT* gene) [46]. Both hyperdopaminergic and hypodopaminergic stimulations have been associated with abnormal functioning of the PFC [47]. The Val158Met polymorphism is the most discussed in scientific literature. According to Caspi et al., homozygosity for the Val allele is more frequently associated with conduct disorder than the Met variant, and it increases the risk of aggressive behavior in young men with attention deficit hyperactivity disorder (ADHD) [48]. Schizophrenic men with at least a Met allele had a risk of violent behavior increased by 50% than men with Val allele in homozygosity [49].

### 4.4. DAT-1

*DAT-1* (also known as *SLC6A3*) encodes the dopamine transporter protein, that regulates the level of intra-synaptic dopamine and the dopamine receptor activation [50]. Some authors found an association between criminal behavior and the number of repeats of *DAT1* [51]. Siblings with a 10-repeat allele had a higher risk of being arrested than the siblings with a 9-repeat allele [52]. The 9–9 genotype is associated with a level of irritability and aggressiveness ten times higher than the 9–10 genotype [53]. Beaver et al. found that the 10-repeat *DAT1* was associated with self-reported delinquent peer affiliation in adolescents with environmental risk factors [51]. In a population of 2380 respondents, Vaske et al. found that a 10-repeat allele increases the risk of alcohol use disorder in young men with alcoholic fathers, while the 9-repeat allele was directly related to the risk of alcohol problems in young females (independently from the condition of the father) [54]. Males with 2- or 3-repeat allele of *MAOA* and 10-repeat allele of *DAT1* in homozygosity had less self-control and an increased risk of criminal behavior [55]. In young males *DAT1* polymorphisms have also been related to promiscuous sexual behavior and criminal activity [56].

### 4.5. DRD2 and DRD4

Polymorphisms of the dopamine receptor D2 (*DRD2*) and dopamine receptor D4 (*DRD4*) are thought to influence human behavior [57]. In 2007, studying a population of 872 males, Beaver et al. found an association between *DRD2/DRD4* and conduct disorder [58]. However, in 2009 the same research group reported that the A1 allele of *DRD2* did not predispose to criminal behavior [59]. Boutwell et al. found that carriers of 7-repeat allele of *DRD4* and A1 allele of *DRD2* were more likely to commit major theft, burglary and gang fights [60]. In another study, Boutwell et al. found that persons with few *DRD4* allele repetitions were less likely to be involved in criminal behavior, meaning that *DRD4* had a positive association with non-abstention in delinquency [61]. Cherepkova et al. reported a higher frequency of *DRD4* 7-repeat allele in criminal offenders and in mixed martial arts fighters without criminal record [62].

### 4.6. GABBR2

The *GABBR2* gene encodes a subunit of the receptor of gamma-aminobutyric acid, the most important inhibitory neurotransmitter in the human brain [63]. Terranova et al. compared two study populations: 47 persons with a criminal record and 139 persons without criminal record. They found that the SNP rs3780428 (situated in the intronic region of the *GABBR2*) had a statistically significant association with the first group (*p* = 0.0067) [18].

### 4.7. BDNF

The *BDNF* gene encodes a neurotrophic factor involved in synaptic plasticity [64]. According to some authors, abnormal synaptic plasticity may increase the risk of aggressive and antisocial behavior [8]. In particular, a polymorphism (Val66Met) has been reported to potentially predispose to crime [65]. Matsushita et al. reported that, despite the genotype and allele distributions of this polymorphism not significantly differing between alcoholic and control cases, alcoholic persons with aggressive behavior and a history of delirium tremens had a significantly higher frequency of AA genotypes [66]. In a sample of 392 subjects with criminal record, Bresin et al. found a relationship between homozygosity for the Val allele of the *BDNF* Val66Met polymorphism and self-injurious behavior. Moreover, the authors did not find any association between these variables and the Met allele [67].

### 4.8. NOS-1

The *NOS-1* gene normally contributes to regulate the inflammatory response [68]. Retz et al. found a statistically significant association between *NOS1* ex1f-VNTR and self-reported impulsiveness (*p* = 0.0052) [69]. Other authors have studied the association between short (182 repeats)/intermediate (192 repeats)/long (204 repeats) variants of *NOS1* Exlf-VNTR and impulsivity or aggressive behavior, finding that in the adults the short variant was associated with attention deficit hyperactivity disorder, cluster B personality disorder and aggressive behavior [67]. Reif et al. found that the short variant of *NOS1 Exlf VNTR* (associated with a low activity of the NOS1 exon lf promoter), was related to reduced activity of the anterior cingulate cortex, a brain area involved in the emotional process [70]. Furthermore, Reif et al. found an association between the short variant of *NOS1* Exlf VNTR and hypoactivation of anterior cingulate cortex [70].

### 4.9. Y Chromosome and Androgens

The role play by the Y chromosome and androgens in criminal behavior is unclear. The presence of *DYS533* allele 14 and *DYS437* allele 14 (two short tandem repeats loci of Y chromosome) increases the risk of aggressive behavior. Conversely, the *DYS437* allele 15 frequency was higher in persons without a history of criminal behavior [71]. Furthermore, some authors found that the short trinucleotide repeat polymorphism in the *AR* (androgen receptor) gene was associated with violent-criminal behavior [72]. Concerning the role of the androgens in criminal behavior, Sjoberg et al. found that those suffering from an antisocial personality disorder had a significantly increased level of testosterone in the cerebrospinal fluid (*p* = 0.008). Moreover, the authors found a significative high level of testosterone in cerebrospinal fluid of persons with *MAOA-L* genotype and diagnosis of antisocial personality disorder (*p* = 0.001) [73].

### 4.10. ZNF

Genes encoding zinc finger proteins (*ZNF*) have also been associated with abnormal and/or criminal behavior. For example, some polymorphisms of *ZNF804A* gene have been associated with drug abuse in European Americans, while Tiihonen et al. reported that, in the brains of violent offenders, ribosomal pseudogene *RPL10P9* and *ZNF132* were upregulated [74,75].

### 4.11. Other Genes

As said, alterations of the serotoninergic and dopaminergic systems may be associated with criminal or violent behavior. Annebrink et al. found a relationship between *ACE I/D* (Angiotensin Converting Enzyme Insertion/Deletion) polymorphism and the CSF (cerebrospinal fluid) level of serotonin and dopamine metabolites, respectively 5-hydroxyindoleacetic acid and homovanillic acid [76]. Regarding the role of the PFC in the development of aggressive behavior, Konar et al. reported an increased expression of the proto-oncogene *C-FOS* in the PFC cortex of animal with hyper-responsive behavior [24]. The *CRHR1* gene is considered to be involved in the responses to stressful events through the activation of the limbic system [77]. Chen et al. found a higher rate of intentional injuries among the carriers of the haplotype H3 (GGA) of the *CRHR1* gene [78]. Furthermore, regarding the *hypothalamic–pituitary–adrenal* axis in the development of criminal behavior, some authors found that some corticotropin releasing hormone binding protein (*CRHBP*) haplotypes (rs10062367G, rs32897T, rs7718461A, and rs7721799G) in association with rs32897 T allele caused an increased risk of robbery behavior (*p* = 0.0213) [79]. The development of anti-social (aggressive) behavior can also be related to epigenetic factors: for instance, an increased methylation of *OXTR* (oxytocin receptor gene) has been associated with callous-unemotional traits and aggressive behavior [80]. Tiihonen et al. found that 30–92% of the hardness of psychopathy is related to the levels of the expression of *RPL109* (Ribosomial Protein L109), *ZNF132* (Zinc finger 132), *CDH5* (Cadherin 5) and *OPRD1* (Opioid Receptor Delta 1) genes [75].

## 5. Conclusions

We started our discussion with the presentation of two cases. In the first, a *5-HTTLPR* SS genotype and a variant of *COMT* (Leu136Leu) in homozygosity were found. In particular, variants of *5-HTTLPR* have been associated with behavioral anomalies (e.g., emotional dysregulation), while the specific *COMT* polymorphism that was found (Leu136Leu) is not well-known in the scientific literature regarding this matter, since the *COMT* variant generally associated with aggressive behavior is Val158Met.

The second case presented a 3-repeat VNTR variant of *MAOA* and a TT genotype for the rs13212041 polymorphism of the *HTR1B* gene, that have been associated with aggressive behavior.

In both the cases, the courts did not consider the genetic findings as evidence of mental incapacity because in scientific literature they have been associated with an abnormal impulse control but none of the crimes was impulsive. Therefore, genetic variations were not considered relevant to the issue of deliberate intent.

As shown by the review of the literature, the relationship between genetic variants and anti-social behavior is often based on case-control studies. Most of the authors agree on the importance of the combination of genetic predisposition and social/family environment and none of the discussed evidence proves that the carriers of these mutations are per se incapable of intentionally committing crimes. However, genetic variants are proved to influence several cognitive processes (like moral judgement and impulse control) and, in combination with neuroradiological evidence, environmental factors and psychiatric disorders, can impair the intent, and thus the willfulness and malice aforethought, to commit a crime [27,81].

As observed by many authors, emphasizing the possible criminogenic significance of some of these variants could be a double-edged sword in the legal field [82]. Indeed, wrongfully believing in a deterministic relationship between some genetic variants and crime would expose the carriers to the risk of social and legal stigma, being considered as latent criminals [83].

In conclusion, albeit genetic analysis in these cases may lead to important evidence, it is always important not to consider genetic variants as the sole determinants of anti-social conduct, and in these cases a comprehensive and multidisciplinary approach should always be adopted [84,85].

## Data Availability

The data presented in this study are available on request from the corresponding author.
